# Prevalence of hyperuricemia and its related risk factors in healthy adults from Northern and Northeastern Chinese provinces

**DOI:** 10.1186/1471-2458-13-664

**Published:** 2013-07-17

**Authors:** Ling Qiu, Xin-qi Cheng, Jie Wu, Jun-ting Liu, Tao Xu, Hai-tao Ding, Yan-hong Liu, Zeng-mei Ge, Ya-jing Wang, Hui-juan Han, Jing Liu, Guang-jin Zhu

**Affiliations:** 1 Department of Clinical Laboratory, Peking Union Medical College Hospital, Peking Union Medical College & Chinese Academy of Medical Science, Beijing 100730, China; 2 Department of Epidemiology, Capital Institute of Pediatrics, Beijing 100020, China; 3 Department of Statistics, Institute of Basic Medical Sciences, Chinese Academy of Medical Sciences & Peking Union Medical College, Beijing 100005, China; 4 Department of Clinical Laboratory, Inner Mongolian People’s Hospital, Hohhot 010017, China; 5 Department of Clinical Laboratory, The 2nd Affiliated Hospital of Harbin Medical University, Harbin 150001, China; 6 Department of Pathophysiology, Institute of Basic Medical Sciences, Chinese Academy of Medical Sciences & Peking Union Medical College, No. 5 Dong Dan San Tiao, Beijing 100005, PR China

**Keywords:** Prevalence, Hyperuricemia, Risk factors

## Abstract

**Background:**

Hyperuricemia (HUA) is a potential risk factor for developing insulin resistance, hypertension, dyslipidemia and cardiovascular disease. Therefore, we studied the prevalence of HUA and associated risk factors in the population of two provinces in northern China.

**Methods:**

Based on the research of Chinese Physiological Constant and Health Conditions conducted in 2008–2010, we enrolled 29,639 subjects in a randomized, stratified study in four sampling areas in Heilongjiang Province and the Inner Mongolia Autonomous Region. We collected 13,140 serum samples to determine biochemical indicators including uric acid(UA), glucose, blood lipids, liver function, and renal function, and finally a representative sample of 8439 aged 18 years and older was determined. We also defined and stratified HUA, hypertension, diabetes, obesity and lipid abnormalities according to international guidelines.

**Results:**

There were significant differences in the UA levels between different genders and regions. The total prevalence of HUA is 13.7%. Men had a higher prevalence of HUA than women (21% vs. 7.9%; P < 0.0001). As age increased, HUA prevalence decreased in men but rose in women. The suburbs of big cities had the highest HUA prevalence (18.7%), and in high-prevalence areas the proportion of women with HUA also increased. A stepwise logistic regression model was used to filter out twelve HUA risk factors, including age, gender, residence, hypercholesterolemia, hypertriglyceridemia, impaired fasting glucose, hypertension, obesity, abdominal obesity, CKD, drinking and sleeping. After adjusting for these factors, the odds ratio of HUA was 1.92 times higher in men than in women. Compared with agricultural and pastoral areas, the odds ratio of having HUA was 2.14 for participants in the suburbs of big cities and 1.57 in the center of big cities.

**Conclusions:**

The prevalence of HUA is high in northern China. The differences in HUA prevalence by geographic region suggested that unbalanced economic development and health education, therefore HUA prevention measures should be strengthened to improve quality of life and reduce health care costs.

## Background

Uric acid is the final product of purine metabolism in humans. However, the pathophysiology of hyperuricemia (HUA) or elevated serum uric acid (SUA) levels is not clearly understood. It has been reported that 18.8% of the patients with HUA developed into gout in a 5 year follow-up [1]. For years, HUA has been thought to be the same as gout, but elevated SUA levels have now been identified as a potential risk factor for developing insulin resistance, hypertension, dyslipidemia and cardiovascular diseases which are characterized as the so-called metabolic syndrome [2-6]. It has also been reported that prevalence of the metabolic syndrome increases substantially with increasing levels of serum uric acid [7]. Although SUA levels are usually higher in males than in females, there is a noted increase in SUA levels in both sexes with increasing age [8].

The prevalence and incidence of HUA in the world population have steadily increased over the past 40 years [9]. With rapid economic development and prolonged life expectancy, study of the HUA in China and other developing countries has become even more important. Studies of the prevalence of HUA and its relationship with metabolic syndrome in healthy Thai adults suggested that the prevalence of HUA was 10.6% in the overall Thai population, and up to 24.4% among those in Bangkok. In addition, a direct relationship between the level of uric acid and the prevalence of metabolic syndrome was observed [10,11]. In the study of prevalence of HUA and its association with antihypertensive treatment in Taiwanese hypertensive subjects, Chung-Sheng Lin et al. found hypertensive subjects had a higher prevalence of HUA [12]. Several large surveys have yielded prevalence estimates for HUA in the Chinese mainland, mainly among populations of coastal cities [13,14]. However, little information is available about the HUA prevalence in areas inhabited by Chinese ethnic minorities. Furthermore, most of the studies about HUA prevalence in China concerned the eastern, especially in the urban areas and these populations lack of representativeness [15], therefore, the cohort study with larger sample is necessary. Besides, currently there is little data available on the differences in HUA prevalence by geographic region.

The main objective of this study was to estimate the trends of SUA levels and prevalence of HUA based on the recent, nationally representative samples of Chinese men and women (the Chinese Physiological Constant and Health Conditions (CPCHC) 2008–2010). We also evaluated associations of HUA with metabolic syndrome in this population.

## Methods

### Study population

The population-based cross-sectional survey, Chinese Physiological Constant and Health Condition (CPCHC), was conducted during 2008 and 2010. Our research used the samples from Heilongjiang Province (2008) and Inner Mongolian Autonomous Region (2010) in this survey. To maximize the accuracy of prevalence estimates, subjects were selected using a random, multistage cluster sampling scheme in four regions of each province selected according to administrative and economic levels. The four regions of Heilongjiang and Inner Mongolia are Harbin city, Harbin suburbs, Mudanjiang, Hailin and Hohhot, Ulanqab City, Xilin Gol League, Bayannur, which belong to big cities, suburb of big cities, small and medium-sized cities and rural and pastoral areas respectively according to population and economics. Written informed consent was obtained from each participant before data collection. The protocol was approved by the Institutional Review Board of the Institute of Basic Medical Sciences, Chinese Academy of Medical Sciences.

### Inclusions and exclusions

The 2008–2010 CPCHC samples included 29,639 apparently healthy participants. Those who suffered from systemic disease involving diabetes mellitus, hypertension or other cardiovascular, renal, gastro-intestinal, pulmonary disease or cancer were excluded. Moreover, participants taking any medication known to affect carbohydrate and lipid metabolism were also excluded. Of the total number of participants, 44.3% (n = 13,140) were selected randomly to complete blood testing. After excluding individuals aged < 18 years, where the important variables was missing, and the results of biochemical tests exceeded four times the standard deviation of the sample, our final sample size was 8,439 adults (3,725 men and 4,714 women), of which 5,859 were Han Chinese, 954 were Korean-Chinese, 1,292 were Mongolian-Chinese, and 334 were of other ethnicity.

### Data collection and anthropometry

A standard questionnaire was used to obtain demographic characteristics (age, gender, and ethnicity), socioeconomic data (education level, marital status, and occupation), lifestyle, past medical history, and lifestyle risk factors (diet, exercise, smoking, and drinking).

Body weight was measured to the nearest 0.1 kg on a calibrated beam scale and height was measured to the nearest 0.1 cm barefoot in triplicate using a wall-mounted stadiometer. Body mass index (BMI, an index for overall obesity) was calculated as body weight (in kilograms) divided by height (in square meters). Waist circumference (WC) was measured midway between the lower rib margin and the iliac crest at the end of a gentle expiration. Blood pressure was measured after the participant had rested quietly for at least 10 minutes, using an electric sphygmomanometer (OMRON, HEM-7000). Blood pressure was measured three times and the measurements were averaged. When subjects were found to be hypertensive, a physician review was performed to exclude secondary hypertension. All personnel who participated in data collection and anthropometry were trained medical personnel, and all devices were calibrated.

### Laboratory measurements

All procedures were performed following a 9–12 hour overnight fast and all subjects were told to take bland diet before blood testing. Blood was drawn from the antecubital vein of the arm. Total cholesterol (TC), triglycerides (TG), high-density lipoprotein cholesterol (HDL-C) and low-density lipoprotein cholesterol (LDL-C) were measured with a Beckman AU Series Automatic Biochemical Analyzer (Japan), using Sekisui Medical (Japan) reagents. Uric acid (UA), Fasting blood glucose (FBG), creatine, and 17 other biochemical tests were measured with the same instrument, using Beckman AU reagents. All laboratories participating in the survey followed the same internal quality control program that was standardized by the Peking Union Medical College Hospital. Estimated Glomerular Filtration Rate (eGFR)was calculated by the CG/BSA formula [16].

### Definition or classification of HUA and other metabolism dysfunction

HUA was defined as SUA ≥ 416.4 μmol/L (male) or SUA ≥ 356.9 μmol/L (female). Blood pressure was classified as normotensive (SBP < 120 mmHg and DBP < 80 mmHg), pre-hypertensive (SBP: 120–139 mmHg and/or DBP: 80–89 mmHg) and hypertensive (SBP ≥ 140 mmHg and/or DBP ≥ 90 mmHg) by the Seventh Report of the Joint National Committee on the Prevention, Detection, Evaluation, and Treatment of High Blood Pressure (JNC-7). Body mass index (BMI: kg/m^2^) was calculated from measured weight and height. BMI was classified as underweight (< 18.5 kg/m^2^), normal (18.5–25 kg/m^2^), overweight (25–30 kg/m^2^) and obese (> 30 kg/m^2^) by WHO criteria. Abdominal obesity was defined as WC ≥ 90 cm (male) or WC ≥ 80 cm (female) by IDF consensus. The American Diabetes Association criteria was used to classify FBG as normal glucose (FBG < 5.6 mmol/L), impaired fasting glucose (IFG) (FBG ≥ 5.6 mmol/L ≤ FBG < 7.0 mmol/L), and diabetic (FBG ≥ 7.0 mmol/L). Dyslipidemia was classified according to ATP III, TG: Normal < 1.69 mmol/L, Borderline high 1.69–2.26 mmol/L, High 2.26–5.65 mmol/L, Very high ≥ 5.65 mmol/L; TC: Desirable < 5.17 mmol/L, Borderline high 5.17–6.24 mmol/L, High ≥ 6.24 mmol/L; HDL-C: High 1.56 mmol/L, Optimal 1.03–1.56 mmol/L, Low <l.03 mmol/L; LDL-C: Optimal <2.59 mmol/L, Near optimal 2.59–3.38 mmol/L, Borderline high 3.38–4.16 mmol/L, High 4.16–4.94 mmol/L, Very high ≥4.94 mmol/L.

### Statistical analysis

Epidata 3.2 was used for data entry and verification. Data were entered twice by two different people, and inconsistencies were checked with the original records. SAS 9.2 was used for data processing and statistical analysis. For univariate analyses, SUA levels and the prevalence of HUA were described by gender, age and regional groups. Hyperuricemia was considered as the outcome variable, and 7 demographic indicators, 18 habits (including smoking, drinking, labor work, exercise, diet habit, sleeping, and sitting time etc.), 37 parameters on health status (including medical history, sub-health state) and 9 metabolic markers obtained through physical examination and biochemical tests were set as predictor variables. In multivariate analyses, factors that may affect uric acid or HUA were introduced into a logistic regression model. Sub-health and past medical history factors were not included in the model because of a large amount of missing data. The main factors include demographic information, metabolic indicators, and behavioral factors of life including eating habits, physical exercise, smoking and drinking status, sleep time and other factors. The impact of sub-health and medical history on HUA was confirmed by univariate analysis and expert judgment. Mean ± standard deviation was used to describe the levels of uric acid, which is continuous variable with a normal distribution. Prevalence (%) indicates the percentage of healthy Chinese men and women with a condition at the time of data collection. Quantitative indicator comparison was conducted by t-test, and analysis of variance (ANOVA) with the Student-Newman-Keuls (SNK) post-test was used for group comparisons. Results between men and women were compared using the χ2 test. Qualitative data were compared by chi-square partitioning to allow α correction; P < 2α / n (n - 1) was considered statistically significant. The research applied binary multi-factor and non-conditional logistic regression models with stepwise regression. The entering criterion for the logistic regression models was 0.05, and the exclusion criterion was 0.10, to obtain the optimal model for the evaluation of the risk factors for HUA. Odds ratios (OR) and 95% confidence intervals (CI) were calculated to study the associations between various risk factors and HUA. P <0.05 was considered statistically significant.

## Results

### Distribution of uric acid levels

In men, the average uric acid level over the age of 55 was lower than that under 55, but there were no statistically significant differences between average uric acid levels aged 18–24, 25–34, 35–44, 45–54 years and aged 55–64, 65–74, over 75 years respectively (Table 1). In women, the average uric acid levels gradually increased over 45 years of age. Uric acid levels in all age groups were significantly higher in men than that in women (P <0.01), and the total mean uric acid levels in men was higher than that in women (357.7 μmol/L versus 263.2 μmol/L, P < 0.0001) (Table 1 and Additional file 1: Figure S1). Among four different regions, uric acid levels in suburbs of big cities were highest in men, women and the entire study population (Table 1). Participants from small and medium-sized cities and agricultural and pastoral areas have lower average uric acid levels (294.5 μmol/L and 295.6 μmol/L) than that from the center of big cities and suburbs of big cities (309.2 μmol/L and 325.7 μmol/L, P < 0.05). There were no significant differences between uric acid levels in participants from small and medium-sized cities and those from rural and pastoral areas (Table 1). In different ethnic groups, there were significantly differences between uric acid levels in women and the entire study population but no statistical significance in men by SNK test.

**Table 1:** Distribution of uric acid levels by age, sex, region and ethnic

	**Male**		**Female**		**Total**	
	**N**	**M±SD(μmol/L)**	**N**	**M±SD(μmol/L)**	**N**	**M±SD(μmol/L)**
Age group						
18-24 yrs	548	363.7±76.0^a^	684	264.9±56.4^a *^	1232	308.8±82.1
25-34 yrs	584	372.9±79.2^a^	746	247.9±56.7^b *^	1330	302.8±91.7
35-44 yrs	756	364.5±77.3^a^	944	249.5±56.0^b *^	1700	300.5±87.5
45-54 yrs	808	358.4±80.6^a^	1053	263.7±61.2^a *^	1857	304.7±84.5
55-64 yrs	511	334.1±77.4^b^	753	273.5±64.3^a *^	1264	298.0±75.9
65-74 yrs	414	340.1±83.2^b^	454	291.1±73.3^c *^	868	314.4±81.8
> 75 yrs	104	341.4±77.1^b^	80	296.5±84.5^c *^	184	321.9±83.2
Total	3725	357.7±81.6	4714	263.3±62.6^*^	8439	305.0±85.6
Region						
Center of big cities	812	363.1±75.8^a^	1111	269.8±63.3^a *^	1923	309.2±82.8^a^
Suburbs of big cities	778	374.5±78.2^b^	953	285.8±65.0^b *^	1731	325.7±83.8^b^
Small and medium cities	876	347.2±77.9^c^	1068	253.4±55.1^c *^	1944	295.6±81.1^c^
Agricultural and pastoral areas	1250	348.6±82.5^c^	1582	251.8±61.1^c *^	2832	294.5±86.0^c^
Ethnic group						
Han	2662	360.1±81.9^a^	3197	267.8±62.1^a *^	5859	309.7±85.2^a^
Mongolian	532	351.0±76.1^a^	760	247.8±60.8^b *^	1292	290.3±84.5^b^
Korean	387	348.3±89.7^a^	567	257.1±66.0^c *^	954	294.1±88.6^b^

^a b c^: same letter showed that no difference was found between two age groups, two regions or two ethnic groups of a same gender with the SNK test.

^*^T test results of comparison with men, P < 0.01.

*M* mean and *SD* standard deviation.

### Prevalence of hyperuricemia

**Figure 1:**
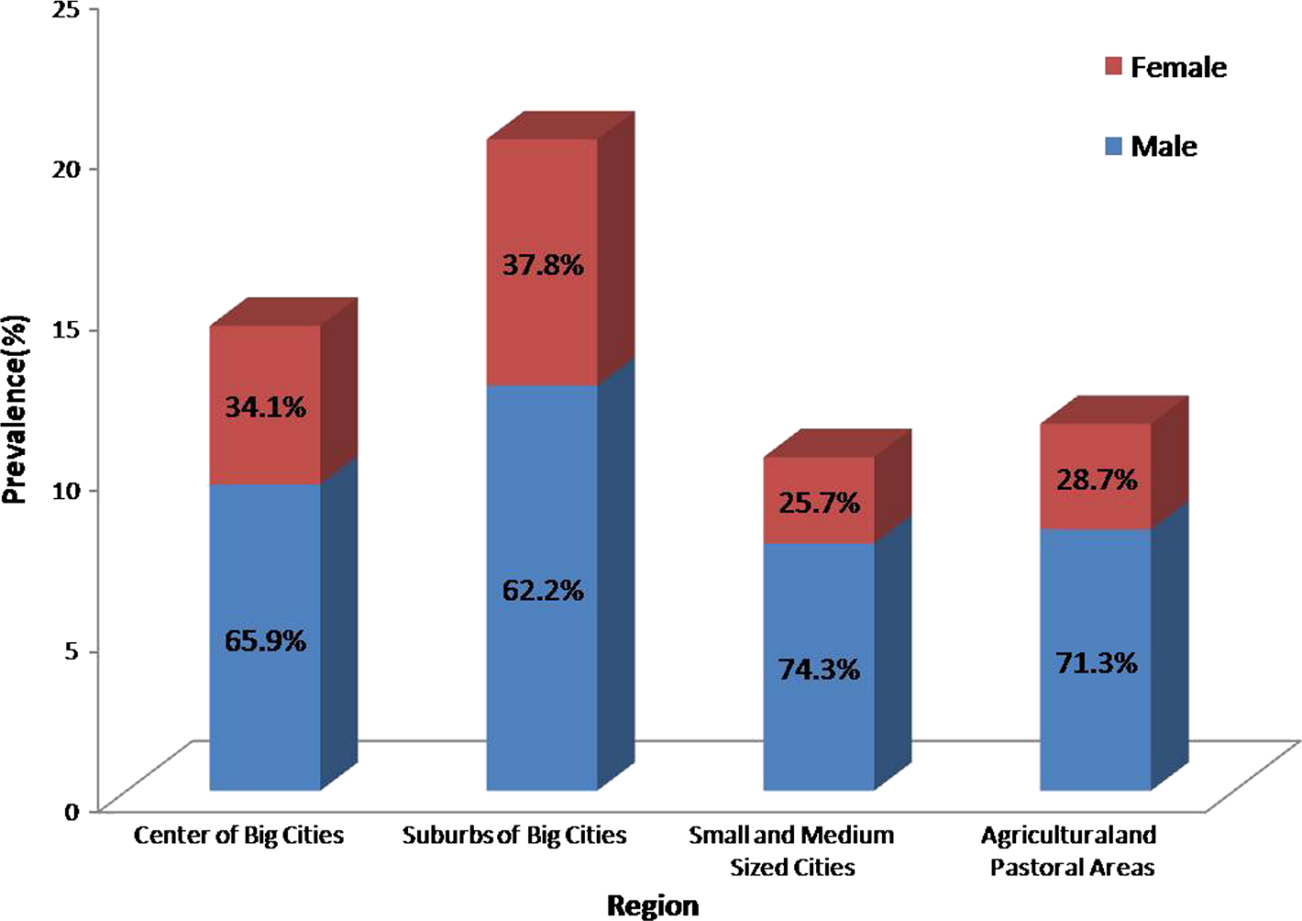
The proportion of male and female in areas with different HUA prevalence.

As shown in Table 2, the total prevalence of HUA is 13.7%. Men had a higher prevalence of HUA than women (21% vs. 7.9%; P < 0.0001). In men, the HUA prevalence decreased with increasing age. In women, HUA prevalence increased in those over 45 years old. In participants over 65 years old, HUA prevalence in men and women was similar (P > 0.05). In those over 75 years old, the prevalence in women was higher than men, but not significantly higher (P > 0.05) (Table 2). In different regions, the prevalence of HUA in men was significantly higher than women and the suburbs of big cities have the highest HUA prevalence in male (28.0%), female (14.0%) and total (20.3%). In men, the HUA prevalence in agricultural and pastoral areas (18.5%) versus small and medium-sized cities (17.1%) was not significantly different (P > 0.05). However, HUA prevalence in the center of big cities (22.6%) and suburbs of big cities (28.0%) was significantly higher than agricultural and pastoral areas, as well as significantly higher than small and medium-sized cities. In women, the prevalence tendency of all regions was similar with men (Table 2). It was also found that, in areas with higher HUA prevalence, the proportion of female patients was significantly higher, which were 25.7% and 28.7% in small and medium-sized cities and rural and pastoral areas respectively, while in the metropolitan central area and suburban, the HUA prevalence ratio in female are 34.1% and 37.8% respectively (Figure 1).

**Table 2:** Prevalence of HUA by age and gender

	**Male**		**Female**		**Total**	
	**n**	**Prevalence(%)**	**n**	**Prevalence(%)**	**n**	**Prevalence(%)**
Age group						
18-24 yrs	116	21.2	49	7.2^#^	165	13.4
25-34 yrs	157	26.9	35	4.7^#^	192	14.4
35-44 yrs	177	23.4	48	5.1^#^	225	13.2
45-54 yrs	178	22.0	73	6.9^#^	251	13.5
55-64 yrs	70	13.7	71	9.4^#^	141	11.2
65-74 yrs	70	16.9	77	17.0	147	16.9
>75 yrs	16	15.4	20	25.0	36	19.6
Total	784	21.0	373	7.9^#^	1157	13.7
Region						
Center of big cities	184	22.6	95	8.6^#^	279	14.5
Suburbs of big cities	219	28.0	133	14.0^#^	352	20.3
Small and medium cities	150	17.1	52	4.9^#^	202	10.4
Agricultural and pastoral areas	231	18.5	93	5.9^#^	324	11.4

^#^Chi-square test results compared with men, *P*<0.01.

### Risk factors for HUA

After stepwise logistic regression screening, 12 variables remained in the model. These were gender, area of residence, age group, hypercholesterolemia, hypertriglyceridemia, impaired fasting glucose, hypertension, obesity, CKD, abdominal obesity, drinking, and sleeping time. Pearson goodness of fit showed a good model fitness (P = 0.9896). In the fully adjusted model with these 12 co-variables, the odds of women having HUA was 0.52 (0.42–0.64) times the odds of men (Table 3). Participants in the suburbs of big cities have an odds of HUA 2.14 (1.71–2.68) times that of the agricultural and pastoral areas, while those in the center of big cities have an odds of HUA 1.57 (1.25–1.98) times that of the agricultural and pastoral areas. With an increase in lipid levels, the odds of HUA also increased. Compared with the control group, the odds of HUA in the hypercholesterolemia group was 1.48 (1.12–1.94). Similarly, compared with the control group the odds ratio of HUA in the hypertriglyceridemia group was 4.30 (2.69–6.86). The fasting glucose impairment group had an odds of HUA 1.27 (1.05–1.54) times higher than the odds of the respective control group, while the diabetic group had an odds of HUA 0.59 (0.40–0.86) times of the normal glucose group. The odds of HUA in the hypertensive group was 1.53 (1.18–1.99) times the odds of the normotensive group. The odds of HUA increased along with an increase in obesity; the odds of HUA in the obese group was 2.42 (1.73–3.39) times the odds of the normal weight group. Compared with the respective control group, the odds ratio of the abdominal obesity group 1.82 (1.46–2.26). As the CKD classification increased, the odds of HUA also increased. The CKD3 class had an HUA odds 6.34 (3.96–10.13) times higher than that of the control group. The odds ratio of HUA in participants who drink group was 1.36 (1.11–1.67) times the non-drinking group. The group with less than four hours of sleep had an odds of HUA 2.48 (1.39–4.42) times the control group.

**Table 3:** HUA risk factors in two Northern Chinese provinces

**Factors**	**n**	**%**	**OR**^**a**^	**95% CI**^**a**^
Gender				
Male	784	21.1	1.00	
Female	373	7.9	**0.52**	**0.42-0.64**
Residence				
Agricultural and pastoral areas	324	11.4	1.00	
Small and medium sized cities	202	10.4	0.92	0.72-1.18
Suburbs of big cities	352	20.3	**2.14**	**1.71-2.68**
Center of big cities	279	14.5	**1.57**	**1.25-1.98**
Age group				
25-44 yrs.	417	13.8	1.00	
18-24 yrs.	165	13.4	**2.01**	**1.52-2.64**
45-64 yrs.	392	12.5	**0.56**	**0.45-0.71**
> 65 yrs.	183	17.4	**0.62**	**0.43-0.92**
TC				
TC < 5.17 mmol/L	662	11.6	1.00	
TC ≥ 5.17 mmol/L &TC<6.24 mmol/L	319	15.8	1.04	0.85-1.26
TC ≥ 6.24 mmol/L	176	24.7	**1.48**	**1.12-1.94**
TG				
TG < 1.69 mmol/L	479	8.4	1.00	
TG ≥ 1.69 mmol/L &TG<2.26 mmol/L	207	18.0	**1.76**	**1.39-2.22**
TG ≥ 2.26 mmol/L &TG<5.65 mmol/L	399	28.8	**2.83**	**2.30-3.49**
TG ≥ 5.65 mmol/L	72	42.1	**4.3**	**2.69-6.86**
FG				
Glu < 5.6 mmol/L	717	11.6	1.00	
Glu ≥ 5.6 mmol/L &Glu<7.0 mmol/L	379	20.8	**1.27**	**1.05-1.54**
Glu ≥ 7.0 mmol/L	61	14.9	**0.59**	**0.40-0.86**
HTN				
Normal	211	7.7	1.00	
PreHTN	569	15.3	**1.51**	**1.22-1.87**
HTN	345	20.2	**1.53**	**1.18-1.99**
Obesity				
Normal	426	9.1	1.00	
Overweight	540	20.0	**1.65**	**1.33-2.05**
Obesity	150	30.1	**2.42**	**1.73-3.39**
CKD				
eGFR≥90 ml/(min·1.73 m^2^)	754	12.7	1.00	
eGFR≥60 ml/(min·1.73 m^2^) & eGFR<90 ml/(min·1.73 m^2^)	291	14.6	**1.82**	**1.42-2.33**
eGFR≥30 ml/(min·1.73 m^2^) & eGFR<60 ml/(min·1.73 m^2^)	86	30.0	**6.34**	**3.96-10.13**
eGFR<30 ml/(min·1.73 m^2^)	5	83.3	**55.87**	**6.10-511.48**
Abdominal obesity				
Normal	496	9.5	1.00	
Abdominal obesity	661	20.4	**1.82**	**1.46-2.26**
Drinking				
Non-drinking	578	10.2	1.00	
Drinking	495	22.8	**1.36**	**1.11-1.67**
Quit drinking	33	16.9	1.07	0.65-1.77
Sleeping time				
>8 hours	222	12.4	1.00	
6-8 hours	681	14.1	1.07	0.88-1.31
4-6 hours	144	13.4	1.03	0.77-1.37
<4 hours	24	17.8	**2.48**	**1.39-4.42**

^a^ Bold text indicates significance at P<0.05.

*Abbreviations*: *TC* total cholesterol, *TG* triglyceride, *FG* fasting glucose, *HTN* hypertension, *CKD* chronic kidney disease.

A stepwise logistic regression model was used to estimate odds ratios (ORs) with 95% confidence intervals (CIs) and all other factors were adjusted when estimate odds ratios (ORs) with 95% confidence intervals (CIs) of each variable.

Odds ratios for various risk factors were also calculated separately for men and women (Additional file 1: Table S1 and Table S2). After stepwise logistic regression screening for men, 13 variables remained in the model. These were residence, work status, age group, cholesterol level, triglycerides, fasting glucose, obesity, CKD, abdominal obesity, diet, sleep time, drinking, and physical exercise. The fully adjusted model indicates that abdominal obesity, hypertriglyceridemia and less sleep time are associated with higher odds of HUA. Oppositely, bland diet group has less odds of HUA. For women, eight variables remained in the model: age group, area of residence, triglycerides, low-density lipoprotein cholesterol, high blood pressure, obesity, CKD, and abdominal obesity. Menopause status was included as a possible co-variable, but was not selected by the stepwise regression for inclusion in the final model. Unlike the general population and men, impaired fasting glucose and total cholesterol level were not selected for inclusion into the model for women. Compared with LDL-C < 2.59 mmol/L, the odds ratio of HUA of LDL-C > 3.38 mmol/L was elevated. The impact of residence was also significant for women: compared with the odds of HUA of participants living in agricultural and pastoral areas, the odds ratio of HUA in the suburbs of big cities was 3.52 (2.35–5.28) and in the center of big cities was 2.24 (1.47–3.40).

## Discussion

In the current study, two Chinese Northern provinces have relatively high HUA prevalence levels of 13.7% (21.1% in men and 7.9% in women). In the suburbs of big cities with higher standards of living, the HUA levels reach 20.3%, while HUA is only 10.4% in small and medium-sized cities with lower standards of living. To our knowledge, this was the first study to examine the differences in HUA prevalence by geographic region in areas inhabited by Chinese ethnic minorities. Our findings suggest that improved living standards bring about a series of changes in nutrition and metabolism which may have significant impact on HUA. In recent years, dozens of surveys on HUA prevalence in mainland China have been conducted [15]. These surveys may not be representative of the entire Chinese population, because most of the study populations are with specific occupations such as state owned factories and mines laborers, or from specific regions such as coastal areas. Seven of these surveys applied randomized sampling, and these studies showed that the HUA prevalence in mainland China ranges from 9.2% to 25.6% in men and 1.3% to 15.3% in women, with significant differences in different areas [17-23]. Surveys in four regions of the Shandong coastal areas presented HUA prevalence ranges from 5.5% to 18.1% [18,19]. In the developed areas of inland Chengdu, the HUA prevalence is 15.6% [22], while in underdeveloped inland Yan’an City the prevalence is 5.4% [23]. According to our results and other published data, we concluded that the HUA prevalence is related to the level of regional economic development and living standards, not whether it is a coastal area. Of interest, in a big city the lifestyle and eating in the suburbs is similar to the city center, but HUA prevalence is higher in the suburbs, which may be because more health education is received in central urban area.

Similar to other researchers [24], we observed that HUA prevalence in women increased post menopause, which is consistent with estrogen protection and renal function in women gradually decreasing with age. However, menopausal status was not selected in the stepwise logistic regression model, possibly because of missing data (Of all 4714 women, 305 women missed the response to “menopausal status”). In participants aged over 75 years old, the HUA prevalence was higher for women than men. This may be because the decline in renal function of elderly women is faster, resulting in poor HUA buffering capacity. Alternatively, because of a small number of participants in this age group, the estimates presented here may lack reliability. Interestingly, the UA levels are high in young men. These high levels may be associated with high androgen levels in young adults, which promote kidney uric acid re-absorption [25]. Unlike other reports [21], this study concluded that the lowest HUA prevalence for men is found in participants aged 55–64, and possible causes include that Chinese men of this phase are retired. At this age heavy labor and mental work also decrease, along with lifestyle changes and more attention on health. All of these factors could contribute to the relative control of uric acid levels at age 55–64.

Many studies have reported relationships between HUA prevalence and hypertension, high blood sugar, obesity and abnormal lipid metabolism and other metabolic abnormalities [26-28]. In this study, multiple logistic regression results have further confirmed the association between metabolic abnormalities and HUA, and have conducted further stratified analysis on each metabolic abnormality-related indicator. Our results have shown that TG and BMI are the most significant contributing factors to HUA prevalence, and that when TG ≥ 5.65 mmol/L, odds of HUA increase nearly 5-fold compared with TG < 1.69 mmol/L. In addition, obese participants have an HUA prevalence of 27.0%, while normal weight subjects have a prevalence of 8.9%, meaning that obese people have HUA prevalence 3.27 times that of those of normal weight. For traditional vascular disease risk factors like TC and LDL, to reach a certain level results in a small increase in HUA odds. This suggests that the lipids are involved in energy metabolism and can increase purine biosynthesis and catabolism, which can eventually contribute to HUA risk factors. However, hardening of the arteries caused by abnormal cholesterol that could cause kidney changes may not greatly contribute to HUA. Fasting blood glucose impaired patients have an HUA prevalence of 27.1%, but compared with normal blood glucose (19.2%), the difference is not significant. Although the HUA prevalence in high fasting blood glucose patients reaches up to 15.2%, the odds ratio reduces to 0.44, which may be because high fasting glucose subjects have already been diagnosed with diabetes and have already begun treatment. In stepwise multiple regression, we found that elevated blood pressure is a risk factor in women but not men. In addition, HUA prevalence in those pre-hypertensive and hypertensive were twice the prevalence of normotensives. Further study is needed to explore the differences of abnormal blood pressure impact on HUA prevalence in men and women.

In addition to blood pressure, other differences in HUA risk factors in different genders were obtained by stepwise multiple regression. Unique risk factors for women were mainly demographic characteristics such as marital status, while unique risk factors for men were mainly personal habits such as diet habits, smoking, alcohol consumption, and sleep time. This interesting phenomenon may indicate that men have less concern for their health. If this is true, active intervention measures to lower uric acid may be more effective for men. In addition, the study also found that participants from transportation and government agencies had high HUA prevalence (19.7% and 19.2%). A common feature of these two occupations is long periods of sitting. Additionally, it may not be possible for transportation employees to urinate on time; whether this factor could cause the increase of uric acid in renal tubular re-absorption remains to be further explored.

The study had several limitations. The study is based on a cross-sectional survey, which is unable to determine causality or the temporal relationship between metabolism indicators and HUA. In addition, parameters including some lifestyle factors and educational status were not filled in the questionnaire due to poor compliance of the local population, which resulted in the correlation of these parameters and hyperuricemia were unable to be analyzed completely because of a large amount of missing data.

## Conclusions

In conclusion, the prevalence of HUA is high in northern China. The differences in HUA prevalence by geographic region presented here suggest that unbalanced economic development and health education may underlie differences in health. The government is suggested to develop programs to effectively control metabolic abnormalities, including HUA. Our research also shows that personal habits are closely associated with HUA. Additional attention should be given to healthy lifestyles and early interventions to control obesity and lipid abnormalities, to effectively reduce HUA risk and avoid the occurrence of gout.

## Competing interests

All the authors declared that there are no competing interests.

## Authors’ contributions

LQ, XQC, JW carried out the experimental design, analysis and interpretation of data, and drafted the manuscript. JTL, TX performed statistical analysis, and drafted the manuscript. YHL and HTD were responsible for the acquisition of data and manuscript revision. ZMG, YJW, HJH and JL participated in the study and the acquisition of data. GJZ conceived the study, participated in its design and coordination, and helped in drafting the manuscript. All authors read and approved the final manuscript.

## Pre-publication history

The pre-publication history for this paper can be accessed here:

http://www.biomedcentral.com/1471-2458/13/664/prepub

## Supplementary Material

Additional file 1: Figure S1 The relationship between serum uric acid (SUA) concentration and hyperuricemia (HUA) prevalence by age group. The bars represent the mean SUA levels in male, female and overall at different age group and the lines represent HUA prevalence. **Table S1**. HUA risk factors in male participants. **Table S2**. HUA risk factors in female participants. Click here for file
