# Supplementary material for: Prevalence of hyperuricemia and its related risk factors in healthy adults from Northern and Northeastern Chinese provinces
Source: BMC Public Health. 2013 Jul 17;13:664. doi: 10.1186/1471-2458-13-664 (PMC3722003; doi:10.1186/1471-2458-13-664)
Supplement: Additional file 1: Figure S1 — The relationship between serum uric acid (SUA) concentration and hyperuricemia (HUA) prevalence by age group. The bars represent the mean SUA levels in male, female and overall at different age group and the lines represent HUA prevalence. Table S1. HUA risk factors in male participants. Table S2. HUA risk factors in female participants. [file 1471-2458-13-664-S1.docx]

Supplemental materials

Figure S1. The relationship between serum uric acid (SUA) concentration and hyperuricemia(HUA) prevalence by age group. The bars represent the mean SUA levels in male, female and overall at different age group and the lines represent HUA prevalence.


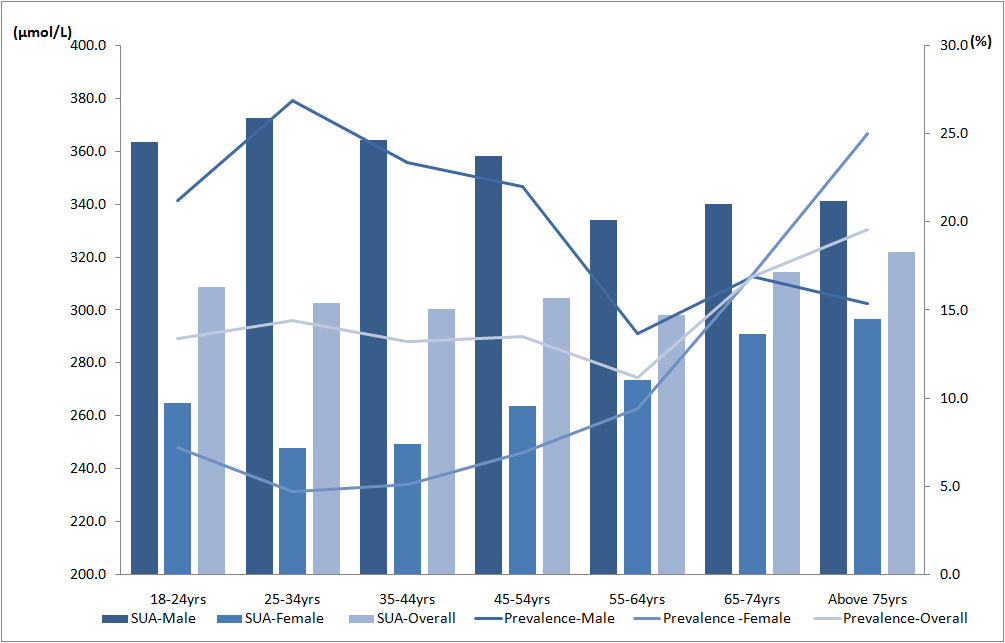


Table S1. HUA risk factors in male participants

| Factor | n | % | OR^a^ | 95%CI^a^ |
| --- | --- | --- | --- | --- |
| Residence |  |  |  |  |
| Agricultural and Pastoral Areas | 231 | 18.5 | 1.00 |  |
| Suburbs of Big Cities | 219 | 28.0 | **1.80** | **1.36-2.39** |
| Small and Medium Sized Cities | 150 | 17.1 | 0.87 | 0.65-1.17 |
| Center of Big Cities | 184 | 22.6 | **1.49** | **1.12-1.98** |
| Work Status |  |  |  |  |
| Unemployed | 67 | 14.8 | 1 |  |
| Labor Work | 100 | 15.6 | 1.12 | 0.73-1.74 |
| Standing | 146 | 22.2 | 1.29 | 0.86-1.92 |
| Sitting | 397 | 24.8 | **1.6** | **1.12-2.30** |
| Age Group |  |  |  |  |
| 25-44 yrs. | 334 | 24.9 | 1 |  |
| 18-24 yrs. | 116 | 21.2 | **1.75** | **1.24-2.47** |
| 45-64 yrs. | 248 | 18.8 | **0.56** | **0.42-0.74** |
| >65 yrs. | 86 | 16.6 | **0.56** | **0.33-0.94** |
| TC |  |  |  |  |
| TC<5.17 mmol/L | 469 | 18.9 | 1.00 |  |
| TC≥5.17 mmol/L &TC<6.24 mmol/L | 202 | 21.7 | 1.01 | 0.79-1.28 |
| TC≥6.24 mmol/L | 113 | 35.7 | **1.55** | **1.09-2.21** |
| TG |  |  |  |  |
| TG<1.69 mmol/L | 299 | 13.5 | 1.00 |  |
| TG≥1.69 mmol/L &TG<2.26 mmol/L | 131 | 24.1 | **1.62** | **1.20-2.17** |
| TG≥2.26 mmol/L &TG<5.65 mmol/L | 295 | 35.2 | **2.93** | **2.28-3.77** |
| TG≥5.65 mmol/L | 59 | 48.0 | **5.06** | **2.98-8.58** |
| FG |  |  |  |  |
| Glu<5.6 mmol/L | 485 | 19.2 | 1.00 |  |
| Glu≥5.6 mmol/L &Glu<7.0 mmol/L | 265 | 27.1 | 1.23 | 0.98-1.56 |
| Glu≥7.0 mmol/L | 34 | 15.2 | 0.42 | 0.25-0.70 |
| Obesity |  |  |  |  |
| Normal | 260 | 14.5 | 1.00 |  |
| Overweight | 379 | 26.7 | **1.69** | **1.28-2.22** |
| Obesity | 115 | 41.7 | **2.71** | **1.78-4.12** |
| CKD |  |  |  |  |
| eGFR>90ml/(min·1.73m^2^) | 552 | 22.2 | 1.00 |  |
| eGFR≥60ml/(min·1.73m^2^)&eGFR<90ml/(min·1.73m^2^) | 170 | 17.1 | **1.44** | **1.06-1.98** |
| eGFR≥30ml/(min·1.73m^2^)&eGFR<60ml/(min·1.73m^2^) | 43 | 30.5 | **4.33** | **2.27-8.28** |
| eGFR<30ml/(min·1.73m^2^) | 1 | 100.0 | - | - |
| Abdominal Obesity |  |  |  |  |
| Normal | 369 | 15.5 | 1.00 |  |
| Abdominal Obesity | 415 | 30.9 | **1.63** | **1.24-2.14** |
| Diet habit |  |  |  |  |
| Non-Bland Diet | 645 | 22.2 | 1.00 |  |
| Bland Diet | 67 | 15.4 | **0.64** | **0.45-0.91** |
| Sleep Time |  |  |  |  |
| >8 hrs. | 146 | 18.9 | 1.00 |  |
| 6-8 hrs. | 476 | 22.0 | 1.1 | 0.85-1.42 |
| 4-6 hrs. | 89 | 20.4 | 1.05 | 0.73-1.53 |
| <4 hrs. | 14 | 25.5 | **3.92** | **1.78-8.66** |
| Drinking |  |  |  |  |
| Non-Drinking | 245 | 16.9 | 1.00 |  |
| Drinking | 470 | 24.9 | **1.37** | **1.10-1.72** |
| Quit Drinking | 32 | 17.7 | 1.14 | 0.68-1.92 |
| Physical Exercise |  |  |  |  |
| Regular Physical Exercise | 328 | 21.9 | 1.00 |  |
| No | 399 | 20.7 | **0.77** | **0.62-0.95** |

^a^ Bold text indicates significance at P<0.05.

Abbreviations: TC, total cholesterol; TG, triglyceride; FG, fasting glucose; CKD, chronic kidney disease.

A stepwise logistic regression model was used to estimate odds ratios (ORs) with 95% confidence intervals (CIs) and all other factors were adjusted when estimate odds ratios (ORs) with 95% confidence intervals (CIs) of each variable.

Table S2. HUA risk factors in female participants

| Factor | N | % | OR^a^ | 95%CI^a^ |
| --- | --- | --- | --- | --- |
| Age Group |  |  |  |  |
| 25-44 yrs. | 83 | 4.9 | 1.00 |  |
| 18-24 yrs. | 49 | 7.2 | **2.21** | **1.36-3.60** |
| 45-64 yrs. | 144 | 8.0 | **0.63** | **0.41-0.97** |
| > 65 yrs. | 97 | 18.2 | 0.7 | 0.37-1.33 |
| Residence |  |  |  |  |
| Agricultural and Pastoral Areas | 93 | 5.9 | 1.00 |  |
| Suburbs of Big Cities | 133 | 14.0 | **3.52** | **2.35-5.28** |
| Small and Medium Sized Cities | 52 | 4.9 | 1.16 | 0.72-1.86 |
| Center of Big Cities | 95 | 8.6 | **2.24** | **1.47-3.40** |
| TG |  |  |  |  |
| TG<1.69 mmol/L | 180 | 5.1 | 1.00 |  |
| TG≥1.69 mmol/L &TG<2.26 mmol/L | 76 | 12.5 | **1.94** | **1.31-2.86** |
| TG≥2.26 mmol/L &TG<5.65 mmol/L | 104 | 18.9 | **2.57** | **1.76-3.76** |
| TG≥5.65 mmol/L | 13 | 27.1 | **3.25** | **1.04-10.17** |
| LDL-C |  |  |  |  |
| LDL<2.59 mmol/L | 107 | 7.9 | 1.00 |  |
| LDL≥2.59 mmol/L &LDL<3.38 mmol/L | 69 | 12.4 | 0.95 | 0.66-1.35 |
| LDL≥3.38 mmol/L &LDL<4.16 mmol/L | 25 | 14.7 | **1.83** | **1.17-2.87** |
| LDL≥4.16 mmol/L &LDL<4.94 mmol/L | 9 | 18.4 | 1.73 | 0.87-3.43 |
| LDL≥4.94 mmol/L | 9 | 20.0 | 2.62 | 0.76-9.02 |
| HTN |  |  |  |  |
| Normal | 79 | 4.1 | 1.00 |  |
| PreHTN | 182 | 9.7 | **2.05** | **1.44-2.92** |
| HTN | 107 | 14.2 | **2.12** | **1.35-3.34** |
| Obesity |  |  |  |  |
| Normal | 166 | 5.8 | 1.00 |  |
| Overweight | 161 | 12.6 | **1.74** | **1.21-2.50** |
| Obesity | 35 | 15.8 | **2.08** | **1.14-3.78** |
| CKD |  |  |  |  |
| eGFR>90ml/(min·1.73m^2^) | 202 | 5.8 | 1.00 |  |
| eGFR≥60ml/(min·1.73m^2^)&eGFR<90ml/(min·1.73m^2^) | 121 | 12.1 | **2.36** | **1.56-3.56** |
| eGFR≥30ml/(min·1.73m^2^)&eGFR<60ml/(min·1.73m^2^) | 43 | 29.5 | **10.53** | **5.20-21.34** |
| eGFR<30ml/(min·1.73m^2^) | 4 | 80.0 | **50.44** | **4.88-521.17** |
| Abdominal Obesity |  |  |  |  |
| Normal | 127 | 4.5 | 1.00 |  |
| Abdominal Obesity | 246 | 13.0 | **2.00** | **1.37-2.93** |

^a^ Bold text indicates significance at *P*<0.05.

Abbreviations: TG, triglyceride; LDL-C, low density lipoprotein cholesterol; HTN, hypertension; CKD, chronic kidney disease.

A stepwise logistic regression model was used to estimate odds ratios (ORs) with 95% confidence intervals (CIs) and all other factors were adjusted when estimate odds ratios (ORs) with 95% confidence intervals (CIs) of each variable.
